# Seroprevalence and correlates of HIV, syphilis, and hepatitis B and C virus among intrapartum patients in Kabul, Afghanistan

**DOI:** 10.1186/1471-2334-8-119

**Published:** 2008-09-17

**Authors:** Catherine S Todd, Malalay Ahmadzai, Faridullah Atiqzai, Suellen Miller, Jeffrey M Smith, Syed Alef Shah Ghazanfar, Steffanie A Strathdee

**Affiliations:** 1 Division of International Health & Cross-Cultural Medicine, University of California, San Diego, La Jolla, California, USA; 2 UNICEF, Afghanistan Office, UNAMA Compound, Kabul, Afghanistan; 3 Ministry of Public Health, Islamic Republic of Afghanistan, Kabul, Afghanistan; 4 Womens Global Health Imperative, University of California, San Francisco, San Francisco, California, USA; 5 Jhpiego Corporation, Baltimore, Maryland, USA

## Abstract

**Background:**

Little current information is available for prevalence of vertically-transmitted infections among the Afghan population. The purpose of this study is to determine prevalence and correlates of human immunodeficiency virus (HIV), syphilis, and hepatitis B and C infection among obstetric patients and model hepatitis B vaccination approaches in Kabul, Afghanistan.

**Methods:**

This cross-sectional study was conducted at three government maternity hospitals in Kabul, Afghanistan from June through September, 2006. Consecutively-enrolled participants completed an interviewer-administered survey and whole blood rapid testing with serum confirmation for antibodies to HIV, *T. pallidum*, and HCV, and HBsAg. Descriptive data and prevalence of infection were calculated, with logistic regression used to identify correlates of HBV infection. Modeling was performed to determine impact of current and birth dose vaccination strategies on HBV morbidity and mortality.

**Results:**

Among 4452 women, prevalence of HBsAg was 1.53% (95% CI: 1.18 – 1.94) and anti-HCV was 0.31% (95% CI: 0.17 – 0.53). No cases of HIV or syphilis were detected. In univariate analysis, HBsAg was associated with husband's level of education (OR = 1.13, 95% CI: 1.01 – 1.26). Modeling indicated that introduction of birth dose vaccination would not significantly reduce hepatitis-related morbidity or mortality for the measured HBsAg prevalence.

**Conclusion:**

Intrapartum whole blood rapid testing for HIV, syphilis, HBV, and HCV was acceptable to patients in Afghanistan. Though HBsAg prevalence is relatively low, periodic assessments should be performed to determine birth dose vaccination recommendations for this setting.

## Background

Testing for human immunodeficiency virus (HIV), syphilis, and hepatitis B surface antigen (HBsAg) in pregnancy and labor is medically indicated to prevent vertical transmission. Prevalence of these infections among the antenatal population may be a reliable indicator of general population prevalence and determinant of vaccination policy [1-3]. The intrapartum period may be the only clinical access point for the antenatal population in limited resource settings. Successful interventions to prevent vertical transmission linked to intrapartum rapid testing have been demonstrated in a variety of limited resource settings [4,5]. One such intervention is birth dose hepatitis B vaccine. Recommendations for birth dose vaccination are determined by antenatal prevalence of hepatitis B e antigen (HBeAg) and to some degree by HBsAg in settings where HBeAg prevalence is unknown [6]. Risk of vertical transmission should not be underestimated in settings where HBeAg is relatively low or assumed to be low; Chauvin *et al. *indicate that even in settings where HBeAg prevalence among mothers is relatively low (< 20%), the number of chronic carriers resulting from lack of birth dose vaccination exceeds 500/100,000, the threshold for instituting other neonatal immunization programs, such as poliomyelitis [7]. In many settings, resources do not exist to assess HBeAg prevalence so vaccination policy may be made on HBsAg prevalence alone.

Afghanistan has an evolving health infrastructure and many barriers remain to accessible care, including ongoing conflict and a shortage of trained providers. Blood donor data is the sole published source of population-based prevalence data. From March through December 2006, the Central Blood Banks in Afghanistan reported prevalence of transfusion-associated infections based on single screening test protocol [8]. For Kabul province, low prevalence of HIV antibody (0.0006%), syphilis-suggestive reaction (VDRL 1.1%), HBsAg (3.9%), and hepatitis C antibody (anti-HCV) (1.9%) was reported, with HBsAg prevalence suggesting intermediate prevalence [[8]; see additional file 1]. For the last three years in Afghanistan, monovalent hepatitis B vaccine has been administered simultaneously with diphtheria-pertussis-tetanus (DPT) vaccine at two, four, and six months of life. In 2005, the DPT completion rate for the Kabul urban area was estimated to be 28.6% [9].

Hepatitis B virus (HBV) prevalence has also been reported among Afghan refugee populations outside of Afghanistan, with higher prevalence noted than that reported among Afghan blood donors [10,11]. Since 2002, the return of 4.8 million refugees, largely from neighboring countries with higher prevalence of HBV and hepatitis C virus (HCV) (e.g. Iran, Pakistan), raises concern that prevalence of hepatitis and other blood-borne infections within the country may increase [11]. Reliable general population prevalence is also needed to inform evolving vaccination and sexually transmitted infection (STI) prevention programs. The purposes of this study were to determine the prevalence and correlates of HIV, syphilis, and hepatitis B and C infection and to model the impact of various vaccination strategies at measured HBsAg prevalence among intrapartum patients in Kabul, Afghanistan.

## Methods

### Setting

Kabul, the capital of Afghanistan, had a population of approximately 2.5 million in 2005, with an estimated facility-based delivery rate for urban areas of 85.2% [9,12]. There are four public maternity hospitals in the city, providing free obstetric care locally and serving as regional referral centers for other facility- or home-based deliveries. At the time of the study, the three hospitals with highest delivery volumes in the previous quarter (Malalai, Rabia Balkhi, and Khair Khana) were included. Prior to enrollment, approval was obtained from the Afghan Ministry of Public Health and University of California, San Diego (Protocol #060083) institutional review boards. All study procedures were followed in accordance with the ethical standards of these review boards.

### Participants

Eligibility for this cross-sectional study was confined to pregnant women admitted in stable medical condition; women with conditions requiring urgent medical intervention (e.g. eclampsia, hemorrhage), imminently delivering (cervical dilation ≥ 8 cm), or unable to provide informed consent were excluded. Potential participants were consecutively evaluated from June to September, 2006. Potential study participants either declining participation or ineligible for participation were enumerated and assessed by age or parity for comparison to enrolled participants.

### Questionnaire Development

Questionnaires were developed with input from a local advisory board of reproductive health specialists in Kabul. Following pilot-testing and board review, the final questionnaire was edited, translated, and back-translated for accuracy. The instrument assessed demographics, socioeconomic status, and time spent outside the country, including prior refugee status. Past medical and obstetric history was queried, including number, site, year, and outcome of prior deliveries and prenatal care for the current pregnancy. Risk factors for sexually transmitted and blood-borne infections, such as drug use or number of therapeutic injections, were also assessed.

### Enrollment Procedures

Study representatives were female medical providers (midwives and physicians) who were not employees of the study hospitals. Study representatives were trained in human subjects' research, questionnaire administration, rapid testing, and pre and post-test counseling. Trained representatives were present in shifts to ensure 24 hour enrollment coverage. All women presenting for admission were assessed for study entry. Following medical evaluation and admission, eligible women were invited to accompany the study representative to a private room for informed consent; all women provided written informed consent prior to enrollment. Consented participants were assigned a study number, unlinked to any hospital identifiers, that was the sole identifier on all study materials.

Participants completed an interviewer-administered questionnaire. Pre-test counseling, whole blood rapid testing with finger stick specimen, and post-test counseling with provision of results were then performed. Following these activities, participants were escorted back to the labor ward. Participants who were too uncomfortable for interview or testing following consent were excused from the study (n = 3); those who were somewhat uncomfortable during interview had the interview suspended and testing performed, with the questionnaire completed after delivery. Free testing was the only compensation provided for participation.

### Patient Testing and Prophylaxis

Rapid testing was performed with Abbott Determine^® ^HIV 1/2, Syphilis TP, and HBsAg (Abbott Diagnostics Japan, Tokyo, Japan) and with Standard Diagnostics Anti-HCV (Standard Diagnostics Laboratories, Kyonggi-do, Korea). A second rapid test (OraSure OraQuick HIV 1/2^®^, OraSure Technologies, Bethlehem, PA) was available for tandem HIV testing, with Western Blot (HIV BLOT 2.2^®^, GeneLabs Diagnostics, Singapore) available for confirmation. *Treponema pallidum *particle agglutination (TPPA) assay (Fujirebio Diagnostics, Malvern, PA, USA) and rapid plasma reagin (RPR) (Spectra Diagnostics, Cairo, Egypt) were available for syphilis confirmation and clinical monitoring. All participants with positive rapid tests underwent intravenous sampling, from which serum was prepared and stored for confirmatory testing.

HBV was confirmed with a second, serum-based rapid test (Standard Diagnostics HBV, Standard Diagnostics Laboratories, Korea), as nucleic acid testing was not available. Use of the Determine^® ^and Standard Diagnostics^® ^rapid tests with sensitivities of 99.0% and 99.0% and specificities of 99.0% and 100.0%, respectively, have a positive predictive value (PPV) of 99.988%, assuming a baseline prevalence of HBsAg of 5.0% [13,14]. HCV antibody was confirmed with RIBA (RIBA 3.0 SIA^®^, Chiron Corporation, Emeryville, CA, USA). All confirmatory testing was performed at the VCT Center in Kabul by trained laboratory personnel.

Participants with any positive rapid test (or two for HIV) were treated as presumptive positives and received intra- or post-partum prophylaxis. Protocols were in place for management of prevention of vertical transmission of HIV or HBV and maternal and neonatal syphilis, following international guidelines [15-17].

### Analysis

Student's T-test and the binomial probability test were used to compare participants and those ineligible or declining entry. Descriptive statistics were generated to further characterize the study population. Prevalence for each infection was calculated with confidence intervals based on the Poisson distribution for anti-HCV and HBsAg. The sample size was sufficient to determine at least a 28% difference in demographic and risk variables between HBsAg-positive and HBsAg-negative participants (P = 80%, two-sided alpha = 0.05). Correlates of HBsAg were assessed with univariable logistic regression. Data were entered and cleaned with EpiData 3.1 (EpiData Association, Odense, Denmark) and analyzed with Stata 8.0 (Stata Corp, College Station, Texas).

### Hepatitis B vaccination modeling

The Hepatitis B Disease Burden and Vaccination Model Version 1.2 from the Centers for Disease Control and Prevention was used to estimate hepatitis cases averted for several vaccination strategies, given measured HBsAg seroprevalence [18]. The model requires input of prevalence of maternal hepatitis B e antigen (HBeAg) and child and adult hepatitis B core antibody (anti-HBc) among those positive for HBsAg, for which there is no data from Kabul. A small study done among Afghan refugees in Iran reported that, of women testing positive for HBsAg, no women had HBeAg [11]. However, due to the small sample size, we elected to use data from other sources for this calculation. India was the country of closest geographic proximity with published HBeAg prevalence among antenatal populations positive for HBsAg, ranging between 7.8% and 18.0%; [19-21] we used the estimate of 12.5% for presumed HBeAg prevalence among women testing positive for HBsAg in the model. The model population was the birth cohort for the maternity hospitals of Kabul for 2006 less the probable neonatal and infant deaths (based on national infant mortality rate, resultant n = 45,090). Estimates of anti-HBc of 30% for children and 60% for adults were used to complete the model, which are identical to those used for a model created by the World Health Organization in development of a HBV vaccination strategy for Afghanistan [22].

## Results

Enrollment comprised 4,452 participants, with 1,179 women ineligible and 153 declining participation. Reasons for ineligibility included imminent delivery (62.9%), emergent operative intervention (22.5%), hemorrhage (7.2%), non-reassuring fetal status (6.7%), or other reasons (e.g. eclampsia, 0.7%). Participants were younger (mean 25.74 vs. 27.70, p < 0.001) and had lower parity (mean 2.09 vs. 3.41, p < 0.001) than those ineligible. Women declining enrollment were of greater parity (mean = 3.06, p < 0.001), but did not differ significantly by age.

Demographic and healthcare access information is displayed in Table 1. Generally, participants were young, originated from Kabul province, and had little or no formal education. Regarding high risk behaviors, no participants reported personal non-medicinal drug use and only 2.0% (n = 90) reported drug use by their spouses, of whom 45.5% stated that their husbands had ever injected drugs. Few participants reported that their spouses had engaged in extramarital sexual contact with either men or boys (0.2%, n = 7) or other women (0.7%, n = 30). Few participants had lived in a refugee camp (6.5%, n = 289) or had spouses that had been incarcerated (5.0%, n = 221). The majority had not received therapeutic injections from either a medical (75.0%, n = 3,333) or non-medical provider (99.1%, n = 4,405) in the last year.

**Table 1:** Descriptive Characteristics of Women Delivering in Government Hospitals between June and September, 2006 in Kabul, Afghanistan (n = 4452).

Variable	Mean	Median	Range
Age	25.74 years	24 years	14 – 48 years
Age at Marriage	19.49 years	18 years	10 – 37 years
Number living in household	7.36	5	1 – 40
Variable	Classification	Number (%)	
Ethnic Group (n = 4444)	Tajik	3158 (71.1%)	
	Pashtun	339 (7.6%)	
	Hazara	923 (20.8%)	
	Other	24 (0.5%)	
Country of Birth (n = 4448)	Afghanistan	4420 (99.3%)	
	Other	28 (0.7%)	
Province of Birth (n = 4422)	Kabul & surrounding provinces	3492 (79.0%)	
	Northern	406 (9.2%)	
	Central	299 (6.8%)	
	Eastern	172 (3.9%)	
	Western	25 (0.6%)	
	Southern	28 (0.6%)	
Lived outside Afghanistan in last 5 years (n = 4348):	Yes	1650 (37.1%)	
Educational Level (n = 4447)	No formal education	2996 (67.4%)	
	1–2 years	179 (4.0%)	
	Finished primary school	404 (9.1%)	
	Some secondary school	326 (7.3%)	
	Finished secondary school	349 (7.9%)	
	Some university	113 (2.5%)	
	Finished university	80 (1.8%)	
Frequency Family Eats Meat (n = 4447)	< Once weekly	361 (8.1%)	
	Once weekly	1900 (42.7%)	
	Twice weekly	1492 (33.6%)	
	> Twice weekly	684 (15.6%)	
Usual Means of Transport (n = 4444)	Public Bus	1848 (41.6%)	
	Taxi	1366 (30.7%)	
	Family Car	1175 (26.4%)	
	On foot	39 (0.9%)	
	Motorcycle	9 (0.2%)	
	Horse cart	4 (0.1%)	
Seen medical provider this pregnancy (n = 3930)	Yes	3930 (88.4%)	
Number of Prior Pregnancies	0	1495 (33.6%)	
	1	719 (16.2%)	
	2	653 (14.7%)	
	3	545 (12.2%)	
	4	364 (8.2%)	
	5	299 (6.7%)	
	≥ 6	377 (8.4%)	
Last Birth Attended (n = 2936)	Yes	2283 (77.3%)	

No participants tested positive for either syphilis or HIV. Prevalence of HBsAg was 1.53% (95% CI: 1.18 – 1.94) and anti-HCV was 0.31% (95% CI: 0.17 – 0.53). There were two false positive HBsAg Determine^® ^rapid tests; the second serum-based rapid test was negative (PPV = 97.1%). There were no participants testing positive for both HBsAg and anti-HCV. The absence or low prevalence of HIV, syphilis, and anti-HCV precluded subsequent analysis of correlates.

### Correlates of HBsAg

Only spousal educational level was associated with HBsAg infection in univariate analysis (OR = 1.13, 95% CI: 1.01 – 1.26). When stratified by level of education, only those women whose husbands had finished university were more likely to have HBsAg, while women whose husbands had no formal education were less likely to have HBsAg (Table 2). There was no significant relationship detected between a positive HBsAg test and any other variable reflecting demographics, health care utilization, or high-risk behaviors for either the participant or her spouse in univariate analysis (data not shown).

**Table 2:** Stratified univariate logistic regression analysis of association between maternal hepatitis B surface antigen and husband's educational level (n = 4452).

Level of Husband's Education (n)	Maternal HBsAg n, (%)	OR, (95% CI)
No formal education (n = 1937)	20, (1.03%)	0.54, (0.32 – 0.91)
1 – 2 years of formal education (n = 97)	1, (1.03%)	0.67, (0.09 – 4.85)
Completed primary school (grade 6) (n = 400)	9, (2.25%)	1.56, (0.77 – 3.17)
Completed some secondary school (n = 505)	12, (2.38%)	1.69, (0.90 – 3.18)
Completed secondary school (n = 905)	15, (1.66%)	1.11, (0.62 – 1.98)
Completed some university (n = 225)	0, (0%)	------
Completed university or higher (n = 309)	11, (2.90%)	2.10, (1.09 – 4.05)

### Hepatitis B Vaccination Modeling

Based on measured prevalence of HBsAg, 1313 cases of chronic HBV would be averted with current vaccination practice (Table 3). Were birth dose vaccination introduced with 85% coverage, perinatal cases would be reduced by 27% (Table 4). However, cases of chronic hepatitis, hepatocellular carcinoma, or death from hepatic disease would not be significantly altered with birth dose vaccination (p = 0.11).

**Table 3:** Hepatitis B acquisition and future burden estimates for infants born in government maternity hospitals with current vaccination programming with the DPT series in Kabul, Afghanistan.

	Number Without Program	Number With Program	Number Prevented	Percent Reduction
A. Disease Events Prevented (Future burden)				
Total HBV infections	27,054	19,735	7,319	27%
Acute symptomatic hepatitis B cases	5,400	3,933	1,467	27%
Chronic HBV infections	4,937	3,625	1,313	27%
HBV-related deaths:				
Acute hepatitis B	23	17	6	27%
Cirrhosis	268	197	71	27%
Hepatocellular carcinoma	170	125	45	27%
Total Deaths	460	338	123	27%
B. Number of Infections by Age at Acquisition of Infection (Future burden)				
Perinatal Infection	117	117	0	0%
Early Childhood Infection	13,410	9,766	3,643	27%
Late Infection	13,527	9,852	3,675	27%
Total Infections	27,054	19,735	7,319	27%
C. Number of Chronic Infections by Age at Acquisition of Infection (Future burden)				
Perinatal Infection	106	106	0	0%
Early Childhood Infection	4,021	2,929	1,093	27%
Late Infection	811	590	220	27%
Total Infections	4,937	3,625	1,313	27%
D. Number of Deaths by Age at Acquisition of Infection (Future burden)				
Perinatal Infection	9	9	0	0%
Early Childhood Infection	362	264	98	27%
Late Infection	89	65	24	27%
Total Deaths	460	338	123	27%
				
Appendix. Input values				
Prevalance HBsAg among women of child bearing age:				1.3
Prevalence HBeAg among HBsAg-positive women of child bearing age:				12.5
Prevalence of any marker of HBV infection among 5 year olds:				30
Prevalence of any marker of HBV infection among 30+ year olds:				60
Estimated Hepatitis B3 coverage:				28.6
Estimated coverage with birth dose hepatitis B vaccine:				0

**Table 4:** Hepatitis B acquisition and future burden estimates for infants born in government maternity hospitals with 85% birth dose hepatitis B vaccine coverage vaccination programming in Kabul, Afghanistan.

	Number Without Program	Number With Program	Number Prevented	Percent Reduction
A. Disease Events Prevented (Future burden)				
Total HBV infections	27,054	19,708	7,346	27%
Acute symptomatic hepatitis B cases	5,400	3,933	1,467	27%
Chronic HBV infections	4,937	3,600	1,337	27%
HBV-related deaths:				
Acute hepatitis B	23	17	6	27%
Cirrhosis	268	195	73	27%
Hepatocellular carcinoma	170	124	46	27%
Total Deaths	460	336	125	27%
B. Number of Infections by Age at Acquisition of Infection (Future burden)				
Perinatal Infection	117	90	27	23%
Early Childhood Infection	13,410	9,766	3,643	27%
Late Infection	13,527	9,852	3,675	27%
Total Infections	27,054	19,708	7,346	27%
C. Number of Chronic Infections by Age at Acquisition of Infection (Future burden)				
Perinatal Infection	106	81	24	23%
Early Childhood Infection	4,021	2,929	1,093	27%
Late Infection	811	590	220	27%
Total Infections	4,937	3,600	1,337	27%
D. Number of Deaths by Age at Acquisition of Infection (Future burden)				
Perinatal Infection	9	7	2	23%
Early Childhood Infection	362	264	98	27%
Late Infection	89	65	24	27%
Total Deaths	460	336	125	27%
E. Number of HBV-Related Deaths in 2000 (Current burden)				
Acute hepatitis B	166			
Cirrhosis	1,130			
Hepatocellular carcinoma	795			
Total Deaths	2,090			
				
Appendix. Input values				
Prevalance HBsAg among women of child bearing age:				1.3
Prevalence HBeAg among HBsAg-positive women of child bearing age:				12.5
Prevalence of any marker of HBV infection among 5 year olds:				30
Prevalence of any marker of HBV infection among 30+ year olds:				60
Estimated Hepatitis B3 coverage:				28.6
Estimated coverage with birth dose hepatitis B vaccine:				85%

## Discussion

This study is among the first to assess prevalence of HIV, syphilis, HBV, and HCV in an obstetric population in modern Afghanistan. HIV was not detected, corroborating low prevalence noted in other Afghan populations [[8] – see additional file 1;[23]]. Prevalence of both HBsAg and HCV were lower than those detected through the Central Blood Bank for Kabul province or among Afghan populations in refugee camps in Balochistan (i.e., the Northwest Frontier province of Pakistan) or in southwest Iran [[8] – see additional file 1; [10,11]]. The associations between HBsAg and therapeutic injections or markers of low socioeconomic status noted in among Afghan refugees and in other settings were not detected in the study population, perhaps because reported therapeutic injections in the last year were fairly uncommon or because low prevalence may have diminished the power to detect some associations [10,24,25]. Given the large number of Afghans repatriating from areas of higher hepatitis prevalence in the last five years, similar assessments should be considered in border areas where former refugee camp denizens often initially reside upon repatriation.

It is unclear why the highest spousal educational level was associated with hepatitis B. This relationship may reflect husbands educated in countries with greater hepatitis B prevalence who, following cessation of civil conflict, returned to Afghanistan, married, and transmitted the infection to their spouse. This relationship is similar to that between HIV and higher educational level in rural populations in Africa, where migration for education and possibly infection occurred [26]. In the Afghan context, adult hepatitis B transmission is likely to be iatrogenic or sexual and may predominantly affect the male population. A study assessing HBsAg seroprevalence among blood donors in Pakistan noted males were more likely to be infected than females; both this study and a study exclusively looking at first-time male blood donors noted that hepatitis B transmission likely occurred through unclean medical or shaving supplies [27,28]. We did not interview or test the spouses, but such an assessment may be indicated.

Screening for HIV and syphilis has been posited as optional in some low prevalence settings as resources expended on screening are not justified by the low number of infections detected [29]. However, analyses indicate that screening for HIV and syphilis is cost effective even in very low prevalence settings [30,31]. We did not detect any cases of HIV or syphilis among participants delivering in government hospitals, suggesting that scarce resources should not be diverted to screening at this time. However, generalization of our findings is limited as only women accessing care in government hospitals were assessed.

In the case of hepatitis B, it is reasonable to consider peripartum testing with birth dose vaccine and parental counseling to ensure completion of the vaccine series, as reported rates of likely vaccination series completion are sub-optimal [9]. Based on international recommendations on birth dose hepatitis B vaccination, modeling did not suggest a significant advantage in changing the current strategy [5,6,32]. However, due to the shifting population dynamics in Afghanistan, routine surveillance should be conducted as birth dose vaccination will be recommended if antenatal prevalence of HBsAg reaches 2% [5,33].

This study has a number of important limitations. First, the population consists only of those women able to access care, likely those of higher socioeconomic status. Because hepatitis has been associated with lower socioeconomic status, the prevalence reported here may underestimate the true prevalence among reproductive-aged women in Kabul. Second, there may have been under-reporting of risky behaviors or other socially-desirable responses in this interviewer-administered survey. Further, as the questionnaire was administered during labor, there may have been response truncation due to discomfort. We attempted to minimize this by conducting all interviews in a private setting with a female interviewer. However, interviewer administration was the only feasible means of data collection, due to low literacy and the prohibitive cost of audio computer-assisted self-interview (ACASI) in this setting. Questionnaires were administered only with the participant's assent; if discomfort was preventing response, the participant was offered the option of resuming the questionnaire following delivery.

## Conclusion

The findings of this study indicate that HIV and syphilis were not present among intrapartum patients tested and that HBsAg prevalence was lower than for other general population groups assessed. A general screening program for HIV and syphilis does not appear cost-effective when the limited available resources are considered. However, HBsAg prevalence indicates hepatitis B is a pertinent public health issue and that routine surveillance among antepartum populations should be considered, particularly should HBeAg prevalence be assessed and found to be high. Future programming should include viral hepatitis in public health service announcements and awareness raising activities, potentially improving vaccination series completion. Intrapartum screening with rapid testing is feasible, acceptable, and suited for periodic surveillance activities as population shifts may introduce measurable changes in viral hepatitis prevalence, resulting in potential change in vaccination policy and practice. However, other models to disseminate testing, vaccine, and health information should also be developed to reach populations that do not access care centers and may be at greater risk for these infections.

## Abbreviations

ACASI: audio computer-assisted self-interview; Anti-HBc: hepatitis B core antibody; DPT: diphtheria, pertussis, and tetanus; HbsAg: hepatitis B surface antigen; HbeAg: hepatitis B e antigen; HBV: hepatitis B virus; HCV: hepatitis C virus; anti-HCV: hepatitis C antibody; HIV: human immunodeficiency virus; STI: sexually transmitted infection; *T.*: *Treponema*; TP: *Treponema pallidum*; VDRL: Venereal Disease Research Laboratories

## Competing interests

The authors declare that they have no competing interests.

## Authors' contributions

CT designed the study, analyzed the data, and assisted with manuscript preparation; MA assisted with study instrument design and pilot-testing, coordinated data collection, and assisted with manuscript preparation; FA assisted with instrument design and data entry; SM assisted with study design and result interpretation; JS assisted with data collection coordination and manuscript preparation; PA assisted with instrument design, data collection, and manuscript preparation; SASG assisted with study design and coordination of data collection; and SS assisted with study design and manuscript preparation. All authors read and approved the final manuscript.

## Pre-publication history

The pre-publication history for this paper can be accessed here:



## Supplementary Material

Additional file 1 **CBBR Report.** The data provided reflect the seroprevalence of the diseases of interest among screened blood donors in Afghanistan in 2006. Click here for file

## References

[B1] al-Owais A, al-Suwaidi K, Amiri N, Carter AO, Hossain MM, Sheek-Hussein MM (2000). Use of existing data for public health planning: a study of the prevalence of hepatitis B surface antigen and core antibody in Al Ain Medical District, United Arab Emirates. Bull World Health Organ.

[B2] Fabiani M, Fylkesnes K, Nattabi B, Ayella EO, Declich S (2003). Evaluating two adjustment methods to extrapolate HIV prevalence from pregnant women to the general female population in sub-Saharan Africa. AIDS.

[B3] Saphonn V, Hor LB, Ly SP, Chhuon S, Saidel T, Detels R (2002). How well do antenatal clinic (ANC) attendees represent the general population? A comparison of HIV prevalence from ANC sentinel surveillance sites with a population-based survey of women aged 15–49 in Cambodia. Int J Epidemiol.

[B4] Lolekha S, Warachit B, Hirunyachote A, Bowonkiratikachorn P, West DJ, Poerschke G (2002). Protective efficacy of hepatitis B vaccine without HBIG in infants of HBeAg-positive carrier mothers in Thailand. Vaccine.

[B5] Department of Vaccines and Biologicals, World Health Organization (2001). Introduction of hepatitis B vaccine into childhood immunization services. Management guidelines, including information for health workers and parents.

[B6] Vryheid RE, Kane MA, Muller N, Schatz GC, Bezabeh S (2000). Infant and adolescent hepatitis B immunization up to 1999: a global overview. Vaccine.

[B7] Chauvin P, Ekra D, Plotkin SA (2002). The cost of not implementing routine neonates immunization programmes in HBsAg high prevalence countries. Vaccine.

[B8] Central Blood Bank (2006). Report of Testing of Blood Donors from March – December, 2006.

[B9] Ministry of Public Health, Indian Institute of Health Management Research/Johns Hopkins Bloomberg School of Public Health (2006). National Risk and Vulnerability Assessment.

[B10] Quddus A, Luby SP, Jamal Z, Jafar T (2006). Prevalence of hepatitis B among Afghan refugees living in Balochistan, Pakistan. Int J Infect Dis.

[B11] Pourkarim MR, Zandi K, Davani NA, Pourkarim HR, Amini-Bavil-Olyaee S (2008). An aberrant high prevalence of hepatitis B infection among Afghans residing in one of the Bushehr refugee camps (Dalaki camp) in the southwest of Iran. Int J Infect Dis.

[B12] Central Statistics Office Annual Report. Kabul-Afghanistan.

[B13] World Health Organization (2001). Hepatitis B surface antigen assays: operational characteristics. Phase 1.

[B14] Standard Diagnostics Rapid test: hepatitis information sheet. http://standardia.com/html_e/mn03/mn03_01_00.asp?intId=2.

[B15] Dao H, Mofenson LM, Ekpini R, Gilks CF, Barnhart M, Bolu O, Shaffer N (2007). International recommendations on antiretroviral drugs for treatment of HIV-infected women and prevention of mother-to-child HIV transmission in resource-limited settings: 2006 update. Am J Obstet Gynecol.

[B16] Mast E, Mahoney F, Kane MA, Margolis HS, Plotkin SA, Orenstein WA, Offitt PA (2004). Hepatitis B vaccine. Vaccines.

[B17] Centers for Disease Control and Prevention (2002). Sexually Transmitted Diseases Treatment Guidelines – 2002. MMWR.

[B18] CDC Advanced Immunization Management AIM e-Learning: Considerations for Introduction of new and under-utilized vaccines. http://aim.path.org/en/vaccines/hepb/assessBurden/model/2.html.

[B19] Prakash C, Sharma RS, Bhatia R, Verghese T, Datta KK (1998). Prevalence of North India of hepatitis B carrier state amongst pregnant women. Southeast Asian J Trop Med Public Health.

[B20] Nayak NC, Panda SK, Zuckerman AJ, Bhan MK, Guha DK (1987). Dynamics and impact of perinatal transmission of hepatitis B virus in North India. J Med Virol.

[B21] Mittal SK, Rao S, Rastogi A, Aggarwal V, Kumari S (1996). Hepatitis B – potential of perinatal transmission in India. Trop Gastroenterol.

[B22] World Health Organization (2005). Introduction of Hepatitis B Vaccine in Afghanistan.

[B23] Joint United Nations Programme on HIV/AIDS Afghanistan. http://www.unaids.org/en/Regions_Countries/Countries/afghanistan.asp.

[B24] Wang CS, Chang TT, Yao WJ, Chou P (2002). Comparison of hepatitis B virus and hepatitis C virus prevalence and risk factors in a community-based study. Am J Trop Med Hyg.

[B25] Khan AJ, Luby SP, Fikree F, Karim A, Obaid S, Dellawala S, Mirza S, Malik T, Fisher-Hoch S, McCormick JB (2000). Unsafe injections and the transmission of hepatitis B and C in a periurban community in Pakistan. Bull World Health Organ.

[B26] Hargreaves JR, Glynn JR (2002). Educational attainment and HIV-1 infection in developing countries: a systematic review. Trop Med Int Health.

[B27] Tareen S, Eslick GD, Kam EP, Byles JE, Durrani AB, Maree SM (2002). High prevalence of hepatitis B virus (HBV) among male blood donors in a developing country: urgent need for systematic screening. Scand J Infect Dis.

[B28] Akhtar S, Younus M, Adil S, Hassan F, Jafri SH (2005). Epidemiologic study of chronic hepatitis B virus infection in male volunteer blood donors in Karachi, Pakistan. BMC Gastroenterol.

[B29] Jensen L, Heilmann C, Smith E, Wantzin P, Peitersen B, Weber T, Krogsgaard K (2003). Efficacy of selective antenatal screening for hepatitis B among pregnant women in Denmark: is selective screening still an acceptable strategy in a low-endemicity country?. Scand J Infect Dis.

[B30] Connor N, Roberts J, Nicoll A (2000). Strategic options for antenatal screening for syphilis in the United Kingdom: a cost effectiveness analysis. J Med Screen.

[B31] Graves N, Walker DG, McDonald AM, Kaldor JM, Ziegler JB (2004). Would universal antenatal screening for HIV infection be cost-effective in a setting of very low prevalence? Modelling the data for Australia. J Infect Dis.

[B32] Kane MA, Rizzetto M, Purcell RH, Gerin JL, Vermin G Hepatitis B control through immunization. Viral hepatitis and liver disease.

[B33] United Nations High Commissioner for Refugees Return to Afghanistan – Still Major Challenges Ahead. http://www.unhcr.org/afghan.html.

