# Supplementary material for: Seroprevalence and correlates of HIV, syphilis, and hepatitis B and C virus among intrapartum patients in Kabul, Afghanistan
Source: BMC Infect Dis. 2008 Sep 17;8:119. doi: 10.1186/1471-2334-8-119 (PMC2557011; doi:10.1186/1471-2334-8-119)
Supplement: Additional file 1 — CBBR Report. The data provided reflect the seroprevalence of the diseases of interest among screened blood donors in Afghanistan in 2006. [file 1471-2334-8-119-S1.pdf]

Positive serologic tests done in the central blood bank & its Provincial branches during the years

1989 – 2005

Most of the following tested are donors but a few of them are referred by hospital or physicians

Years	Test Area	Total Tested	Brucellosis	VDRL	HCV	HBS	HIV	Remarks
1989	Central Blood Bank	Unknown	0	0	0	0	1	Unknown Number of Donors
1994	Central Blood Bank	Unknown	0	0	0	0	1	
1996	Central Blood Bank	5309	20	41	6	71	0	It is mentioned able that during the years 1996-2001 due to internal war the reports were not received regularly
1997	Central Blood Bank	3942	0	8	10	50	0	
1998	Central Blood Bank	4221	0	42	16	97	0	
1999	Central Blood Bank	7964	0	0	13	137	0	
2000	Central Blood Bank	6844	0	0	1	280	1	
2000	Nangarhar	2741	0	4	0	0	0	
2001	Central Blood Bank	6691	0	3	0	114	6	
2001	Nangarhar	3075	0	2	0	0	0	
2001	Qandahar	3915	0	0	0	0	0	
2002	Central Blood Bank	11586	0	1	4	248	1	
2002	Nangarhar	2723	0	0	0	0	0	
2002	Parwan	581	0	0	0	5	0	
2002	Mazar-e-Sharef	964	0	5	0	32	2	
2003	Central Blood Bank	10674	0	32	54	173	6	
2003	Nangarhar	3629	0	23	57	194	1	
2003	Baghlan	890	0	0	0	0	0	
2003	Faryab	877	0	0	0	0	0	
2003	Mazar-e-Sharef	4714	0	0	0	12	0	
2003	Heart	1510	0	0	0	0	0	
2003	Jozjan	861	0	0	0	0	0	
2003	Farah	600	0	0	0	0	0	
2003	Helmand	450	0	0	0	0	0	
2003	Khost	510	0	0	0	0	0	
2003	Ghazni	450	0	0	0	0	0	
2003	Qandarhar	1550	0	0	0	0	0	
2003	Kunduz	1010	0	0	0	0	0	
2004	Central Blood Bank	10514	0	32	80	190	10	
2004	Nangarhar	3525	0	23	90	238	13	
2004	Baghlan	232	0	0	0	9	0	
2004	Faryab	370	0	0	0	0	0	

2004	Qandahar		0	0	0	0	1	
2004	Mazar-e-Sharef	2147	0	0	1	17	0	
2004	Heart	2890	0	0	3	6	2	
2004	Kunar	208	0	0	0	0	0	
2004	Parwan	187	0	0	0	0	0	
2004	Juzjan	1264	0	14	1	77	1	
2004	Logar	888	0	0	5	45	0	
2004	Badakshan	256	0	2	1	18	0	
2004	Farah	0	0	0	0	0	0	
2005	Central Blood Bank	7684	34	47	206	107	7	
2005	Nangarhar	2145	0	0	148	49	9	
2005	Heart	2185	0	1	23	4	1	
2005	Mazar-e-Sharef	1190	0	0	7	1	0	
2005	Baghlan	0	0	0	0	0	0	
2005	Faryab	0	0	0	0	0	0	
2005	Juzjan	584	0	8	42	2	0	
2005	Parwan	168	0	1	8	0	0	
2005	Kunar	11	0	0	1	0	0	
2005	Qandahar	1867	0	0	2	7	3	
2005	Ghazni	210	0	0	0	0	0	
2005	Kunduz	826	0	2	16	38	1	
		24710						
Total		125832	54	291	795	2221	67	

Although according to the significance of the positive HIV/AIDS Cases. In Islamic and backward country like Afghanistan whole information must be kept secret between the doctor and patient, and also on the bases of **MOPH** Advices even the address and personal information of the positive cases of HIV/AIDS should be totally secret. But in spite of the above we categorized the positive HIV/AIDS cases as fallow.

Total Number of the positive HIV/AIDS cases during the year 1989-2005.

Total Number of Female 12	Single	Married	Children	Death
	0	9	3	2
Total Number of Male 55	Single	Married	Children	Death
	23	32	0	2

From the Above Positive Cases, 14 persons were refugee who returned from Pakistan, 8 Persons from Iran, 4 Persons from Saudi- Arabia, 2 Persons from Dubai, 1 from France, 1 from Bulgaria and 37 were from inside Afghanistan. Also it is mentionable that from the total number of the positive HIV/AIDS cases 35 were reported from the central blood bank, 23 cases were reported by Nangrahar Provincial Branch, 3 from hirat, 1 from Mazar - Sharif, 1 from Jozjan 3 from Quandhar and 1 from Kunduz Provincial Branch.

Dear Richard Gordon,

Hope you are doing well, dear sir I want to have your attention to the following points about our running activities and their weakness for prevention of HIV/AIDS in Afghanistan. Hope to improve these weaknesses by your close attention and help of international organizations who are interested in this field.
Dear Sir,
Central Blood Bank (C.B.B), Its branches in Kabul and provincial branches provide, stock and distribute the safe blood for the people who are in need of blood, totally free without any charges. Of course according to our rules and regulation in selective cases blood should be given to the patient by exchange method. It means family members or relatives of the patient should donate their blood to the (C.B.B) and (C.B.B) will provide the needed blood group for the patient. But in emergency cases without any delay or asking for exchange the (C.B.B) provides safe blood for the patient according to the request of the doctor. In any condition before stocking and distributing the blood should be checked for serologic tests (HIV,HBS,HCV,VDRL, Brucellosis) by rapid method because there is no enough facilities for western blot or any other confirming test.

Blood Collecting Sources of (C.B.B)

A. Volunteers Blood Donating students union of Kabul Medical Institute:

During the need of (C.B.B), members of this union help us to provide enough amount of blood for the people who are in need of blood.

B. Blood Collecting Campaign:

According to the need of (C.B.B) once or twice in a month we arrange a blood collecting campaign inside the city by the help of our professional and propagating staff who propagate the significance of the blood collecting campaign, distributing the pamphlet, brochure, Poster and by using the special bus which is a help of France Government to the (C.B.B) and contain four comfortable beds, Generator, Microphone and Loudspeaker, Basin and other necessary equipment, and collect the blood from the usual citizen of Kabul.

C. Family Members of the Patient.

This is the largest group of our donors who donate their blood for their relative who are in need of blood. Of Course in any situation the person will be accepted as a suitable donor only if he/she is fit to the WHO criteria.

NOTE: the mentioned sources of blood are first checked for serologic tests (HIV,HBS,HCV,VDRL and brucellosis)by rapid method in case that the tests are negative the blood is stocked and distributed upon the request of blood.

Anyhow, the tests which are done through the years 1989-2005 in the (C.B.B) and in its provincial branches with a lot of difficulties e.g internal war, lack of strips for serologic tests, kit of anti-sera, blood bag, transfusion set, difficulties in transportation and communication were the main causes due to which our activities record and reports were irregular and dissatisfying. on the other hand the tests are done only on donors blood Who are just a few number of our population and they will not represent the correct number of positive HIV/AIDS cases in Afghanistan. And I think the number of the positive HIV/AIDS cases will be much more than the number which is given here. Because the war of last three decades in Afghanistan which resulted to two million death, one and half million handicaps and five million refugees. But now in these days due to improvement of politico- social condition a lot of refugees have returned to the country, but most of them due to poverty, psychological problems, over crowed livings and addiction are prone to suffer from HIV/AIDS. On the other hand HIV/AIDS doesn't have any effective drugs or vaccine and our all goals and preventive measures for HIV/AIDS are based on awareness and prevention.

On the bases of very recent statistics 85% Afghans don't know about HIV/AIDS and they have not heard any things about it.

Illiteracy, lack of video visual facilities in the rural areas due to lack of electricity, poverty, lack of security, massive and rapid return of refugee to the country, and poor audio visual publicity about HIV are the disappointing factors which can accelerate spread of HIV/AIDS in Afghanistan.

Due to the above factors for effective prevention of HIV/AIDS in Afghanistan and agreement of your Excellency I propose the following points.

1. In the primary phase a research and statistic center for all residence of Kabul to find the correct figures which can help us to estimate Percentage of the positive cases in all over the country.
2. In the second phase Implementation of preventive measure for HIV/AIDS: Informative campaigns, education and dialogue among the people specially in venerable groups (Young Generation, Replace Migrates, Poor people, addictive etc..).
3. Effective management of sexual transmitted diseases because suffered patients are eight times more prone than the others to suffer from the disease.
4. Emphasizing to provide the safe blood (HIV, HBS, HCV, VDRL (-)).
5. Establishment of a center to advice, supervise and treat the positive cases of HIV/AIDS.

In case of possibility and agreement of your Excellency I will provide a short proposal only for the first Phase and submit it to you for the final decision, because I believe that for preventive measures of HIV/AIDS in Afghanistan first cultural values of our community must be kept in mind secondly it should be based on an academic and practical plan to be accepted in our backward country.

Thanks
Your sincerely
Dr. M. N Hassas
M.D & D.C.H
Head of C.B.B
